# Feasibility of a Randomized, Interventional Pilot Clinical Study of Oral Cannabinoids in People with HIV on Antiretroviral Therapy: CTNPT 028

**DOI:** 10.3390/jpm14070745

**Published:** 2024-07-13

**Authors:** Ralph-Sydney Mboumba Bouassa, Judy Needham, Dana Nohynek, Suzanne Samarani, Florian Bobeuf, Lina Del Balso, Natalie Paisible, Claude Vertzagias, Giada Sebastiani, Shari Margolese, Enrico Mandarino, Joel Singer, Marina Klein, Bertrand Lebouché, Joseph Cox, Branka Vulesevic, Alison Müller, Elisa Lau, Jean-Pierre Routy, Mohammad-Ali Jenabian, Cecilia T. Costiniuk

**Affiliations:** 1 Department of Biological Sciences and CERMO-FC Research Centre, Université du Québec à Montréal, Montreal, QC H2X 3Y7, Canada; mboumba_bouassa.ralph-sydney@courrier.uqam.ca (R.-S.M.B.); jenabian.mohammad-ali@uqam.ca (M.-A.J.); 2 Infectious Diseases and Immunity in Global Health Program, Research Institute of the McGill University Health Centre, Montreal, QC H4A 3J1, Canada; suzanne.samarani@muhc.mcgill.ca (S.S.); claude.vertzagias@muhc.mcgill.ca (C.V.); giada.sebastiani@mcgill.ca (G.S.); marina.klein@mcgill.ca (M.K.); bertrand.lebouche@mcgill.ca (B.L.); joseph.cox@mcgill.ca (J.C.); bvule091@uottawa.ca (B.V.); jean-pierre.routy@mcgill.ca (J.-P.R.); 3 Canadian HIV Trials Network, Vancouver, BC V6Z 1Y6, Canada; jneedham@advancinghealth.ubc.ca (J.N.); dnohynek@hivnet.ubc.ca (D.N.); shari.margolese@gmail.com (S.M.); emandarino@rogers.com (E.M.); jsinger@hivnet.ubc.ca (J.S.); amuller@advancinghealth.ubc.ca (A.M.); elau@advancinghealth.ubc.ca (E.L.); 4 Centre for Advancing Health Outcomes, St. Paul’s Hospital, Vancouver, BC V6Z 1Y6, Canada; 5 Department of Medicine, Division of Infectious Diseases and Chronic Viral Illnesses Service, McGill University Health Centre, Montreal, QC H4A 3J1, Canada; florian.bobeuf@muhc.mcgill.ca (F.B.); lina.delbalso@muhc.mcgill.ca (L.D.B.); nathalie.paisible@muhc.mcgill.ca (N.P.); 6 Department of Medicine, Division of Gastroenterology and Hepatology, McGill University Health Centre, Montreal, QC H4A 3J1, Canada; 7 School of Population and Public Health, University of British Columbia, Vancouver, BC V6T 1Z4, Canada; 8 Department of Family Medicine, McGill University Health Centre, Montreal, QC H4A 3J1, Canada; 9 Canadian Institutes of Health Research Strategy for Patient-Oriented Research Mentorship Chair in Innovative Clinical Trials, Montreal, QC H4A 3J1, Canada; 10 Department of Medicine, Division of Hematology, McGill University Health Centre, Montreal, QC H4A 3J1, Canada; 11 Division of Experimental Medicine, McGill University, Montreal, QC H4A 3J1, Canada

**Keywords:** cannabis research, feasibility, barriers, recruitment

## Abstract

Cannabis-based medicines (CBMs) could help reduce systemic inflammation in people with HIV (PWH). In a prospective, randomized pilot study we enrolled participants from August 2021–April 2022 with HIV, aged ≥18 and on antiretroviral therapy and randomly assigned them to cannabidiol (CBD) ± Δ9-tetrahydrocannabinol (THC) capsules for 12 weeks with the primary objective being to assess safety and tolerability. Here we report on timeliness to study initiation, enrolment, compliance and retention rates. The target sample size was not reached. Two hundred and five individuals were approached, and 10 consented and were randomized; the rest refused (reasons: cannabis-related stigma/discomfort; too many study visits/insufficient time; unwillingness to undergo a “washout period” for three weeks) or were not eligible. The age of those randomized was 58 years (IQR 55–62); 80% were male. Only three met all criteria (30% enrolment compliance); seven were enrolled with minor protocol deviations. Compliance was excellent (100%). Eight (80%) participants completed the study; two (20%) were withdrawn for safety reasons (transaminitis and aggravation of pre-existing anemia). Time to study initiation and recruitment were the most challenging aspects. Ongoing work is required to reduce stigma related to CBMs. Future studies should find a balance between the requirements for safety monitoring and frequency of study visits.

## 1. Introduction

Despite the efficacy of antiretroviral therapy (ART) at suppressing HIV replication and preventing progression to AIDS, people with HIV (PWH) have ongoing systemic immune activation and chronic inflammation and suffer from a spectrum of non-AIDS-related comorbidities, even in the context of suppressed HIV viral load [1,2,3,4]. Alleviating chronic inflammation could potentially help to prevent the onset of non-AIDS-related comorbidities in PWH on ART [5,6,7,8]. Furthermore, cannabis is often used by PWH for both medical reasons such as chronic pain, anxiety and depression, but also for recreational reasons, and to improve quality of life [9,10,11,12,13]. The primary cannabinoids in the cannabis plant, delta-9-tetrahydrocannabinol (THC) and cannabidiol (CBD), demonstrate potent anti-inflammatory and anti-fibrotic effects in both experimental models [14,15,16,17,18,19,20,21,22], as well as in human studies [23,24,25]. Studies from observational cohorts have demonstrated that PWH who use cannabis or cannabinoid-based medicines (CBMs) have lower levels of inflammation than non-cannabis users, although a causal relationship cannot be ascertained due to the risk of confounding in such study designs [6].

In order to establish the effect of cannabis on systemic inflammation in PWH, clinical trials are needed. However, there is a lack of preclinical and pharmacokinetic data on CBMs to guide dosing recommendations in PWH7, thereby raising safety and tolerability concerns [26,27]. Between August 2021 (study initiation)–April 2022, we aimed to enroll 26 PWH > 18 years of age on ART for randomization to either CBD ± THC capsules, titrating doses as tolerated, for 12 weeks in a pilot clinical trial to assess safety, tolerability and feasibility [7]. The impacts on safety, tolerability, systemic inflammation and HIV reservoir size were previously published [28,29]. The feasibility of executing a CBM RCT in Canada’s regulatory landscape, where medical cannabis has been legal since 2014 and recreational cannabis has been legal since 2018 [30], has never been previously described. Here we report on timeliness to study initiation, enrolment, compliance and retention outcomes, in addition to feedback from study participants and staff at close-out.

## 2. Materials and Methods

### 2.1. Setting

The Chronic Viral Illness Services (CVIS) clinic at the McGill University Health Center (MUHC)—Glen Site is a multidisciplinary clinic which follows approximately 1700 PWH. Located in Montreal, QC, Canada, it is the referral center for migrants and asylum seekers with HIV infection [31,32]. Given its location within Quebec, the only primarily francophone province in Canada, the province attracts a large number of individuals from Francophone countries, including Haiti and Francophone Africa. Forty percent (40%) of the clients at the clinic are women, most of whom are from HIV-endemic countries [31,32].

### 2.2. Participants

Study inclusion criteria were being 18 years of age or older and having HIV infection with suppressed viral load (VL < 40 copies/mL) for at least 3 years on ART. Using cannabinoid-containing products outside of the study or within 4 weeks of study commencement was an exclusion criterion and participants had to have a negative baseline cannabinoid urine screen. A full list of exclusion criteria was previously published [28,29,30].

### 2.3. Recruitment

Participants were recruited by flagging charts of potentially eligible participants (identified via chart review, based on whether they met inclusion criteria and did not have exclusion criteria, as documented in their chart), informing clinic physicians, nurses and pharmacists about the study through regular emails, posters and verbal discussions. Posters were placed in the clinic waiting room. When individuals expressed interest in the study and desired additional information, a member of study staff discussed with them in detail the study protocol and requirements for participation and potential participants had the opportunity to ask questions.

### 2.4. Ethics

This study was approved by the McGill University Health Centre Research Ethics Board (#2018-4336) and was conducted in conformity with the ICH E6 guidelines for Good Clinical Practice and the principles of the Declaration of Helsinki. Prior to study enrolment, individuals signed a written informed consent form.

### 2.5. Intervention

**Figure 1:**
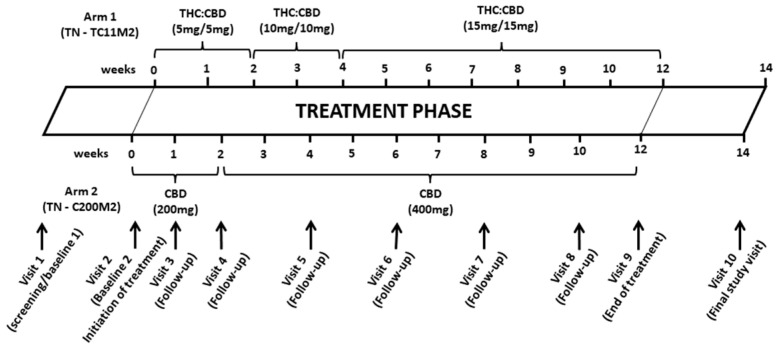
Participants were provided with a recommended titration schedule and titrated up the number of capsules based on their tolerability, a method which has proven successful in other clinical trials.

Following enrolment, participants were randomized 1:1 to receive THC:CBD combination oral capsules (2.5 mg/2.5 mg; TN-TC11M2), or CBD-only oral capsules (200 mg; TN-C200M2) [28,29]. Study capsules were manufactured and provided by Tilray Brands, Inc. (New York City, NY, USA). Participants were provided with a recommended titration schedule (Figure 1) and titrated up the number of capsules based on their tolerability, a method which has proven successful in other clinical trials [33]. We did not include a control group since the creation of a placebo arm would have added significant additional cost, required more time to manufacture the capsules and may have contributed to additional regulatory delays for this initial pilot study. The trial was open. We had considered blinding participants, but blinding in cannabinoid studies is often difficult since participants will often recognize the effects, such as psychoactive or vasoactive effects, which cannabinoids can induce [3].

### 2.6. Follow-Up and Data Collection

The sociodemographic and biological data of the participants were recorded onto data collection worksheets and then entered into electronic case report forms in InForm, a secure web-based electronic data capture platform. After baseline assessment and randomization, participants attended follow-up visits every 2 weeks over the 14-week study period to monitor safety and tolerability [28,29] (Figure 1). Safety and tolerability were assessed by vital signs and adverse event monitoring, as reported by participants and actively sought at each study visit by the study nurse. Biological safety was evaluated by hematology, biochemistry and other clinical, laboratory or other diagnostic tests performed during the course of the study [29]. The toxicity of TN-TC11M2 and TN-C200M2 was assessed using the WHO toxicity scale [29]. Questionnaires measuring quality of life (WHO Quality of Life HIV-BREF (WHOQOLHIV-BREF) and the Euro-Qol-5Dimension (EQ-5D)) and mood (Profile of Mood States (POMS)) were administered at baseline, midway through the study (visit 6) and at the end of the study (visit 9) [7,29]. In total, these took approximately 25 min to complete [7,29,34].

### 2.7. Study Objectives

As previously reported, the primary objective was to evaluate the safety and tolerability of TN-TC11M2 and TN-C200M2 oral capsules in PWH on effective ART, the proportion of participants who were able to complete the study and changes in quality of life and mood questionnaire scores [7,29].

Feasibility outcomes included timeliness to study initiation, consent rate, enrolment and retention. Timeliness to study initiation was measured as time from authorization of protocol V1.0 to site open to recruitment. Ease of enrollment was based on consent rate (number of individuals consented/number of individuals approached). Compliance was assessed based on attendance at study visits (number of appointments scheduled/number of appointments attended and adherence to protocol procedures, such as adherence to titration protocol, and avoidance of exogenous cannabis use based on self-report). Retention was described as the percentage of participants who remained in the study until the primary endpoint was reached (safety and tolerability assessments up to week 12 of treatment). Verbal feedback was solicited from participants and study staff at close-out. There were no thresholds set for feasibility outcomes a priori.

In addition to changes in levels of inflammatory markers, we examined changes in markers of gut mucosal integrity and immune cell subsets. To describe the impact on the HIV reservoir, we measured HIV DNA/RNA in circulating CD4 T-cells and sperm. Reduced gut mucosal integrity is usually associated with higher markers of inflammation and cell activation in PWH. A higher HIV reservoir may also be associated with higher HIV reservoir size. These findings were previously published [28].

## 3. Results

**Figure 2:**
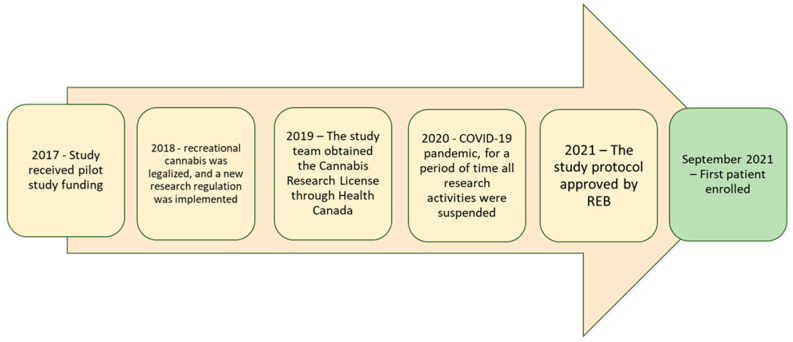
Time to study initiation. Funding was first received in 2017. The first participant was recruited in September 2021.

A study timeline is depicted in Figure 2. The first form of financial support was obtained in 2017. In 2018, Canada implemented the Cannabis Act, legalizing the use of cannabis for recreational purposes and enabling individuals to purchase and possess cannabis without a medical prescription. Along with this change in legislation came the need for researchers to possess a Cannabis Research License through Health Canada, which required 11 months to obtain. In 2019, the Cannabis Research License was obtained.

The COVID-19 pandemic, beginning in 2019, resulted in a near-complete shut-down of all research activities except those directly related to COVID-19 for approximately 6 months. When non-COVID-19 research was permitted to resume, changes were required to our study protocol and informed consent forms, prior to the study to initiation, to enable some tasks to be completed remotely, via telephone, in order to minimize the need for participants to come to our hospital-based clinic. This process took approximately 3 months. Furthermore, the supplier informed us that one of the products we had initially intended to use (capsules containing THC:CBD in a 1:9 ratio) failed a quality control test and there were concerns regarding its stability. To manufacture a new batch of capsules and have them undergo quality control checks would have taken several additional months. Therefore, we opted to use a capsule of 200 mg CBD only, which was already available and in use in other research studies. However, this change also required a change in our protocol and informed consent, and approvals from both our institutional REB and Health Canada (HC), resulting in additional delays to study initiation.

During the study time period, the expiration of our study capsules (shelf live 9 months) was approaching. Although the plant form of cannabis does not have a known “expiration date”, the stability of the manufactured capsules was set at 12 months. However, by the time the capsules are manufactured, complete quality control checks and are shipped to the research site, often 3 months has passed, leaving sites a 9-month window to recruit and distribute capsules and for participant consumption before expiration. Furthermore, during the course of our study, Tilray merged with another company and announced the closure of their manufacturing facility in Nanaimo, British Columbia, Canada, thus the production of cannabinoid capsules using the exact same protocols with which we initiated the study could not be guaranteed. If we had wanted to prolong the period of study recruitment and manufacture more capsules, this would require a new HC application as the product would essentially be new. The study opened to enrolment in Aug 2021 and closed to enrolment in April 2022. The site was not able to enrol any participants in March and April 2022. The study was closed to enrolment at the end of April due to product expiry. The main barriers and facilitators to the study are summarized in Table 1.

### 3.1. Consent Rate

**Figure 3:**
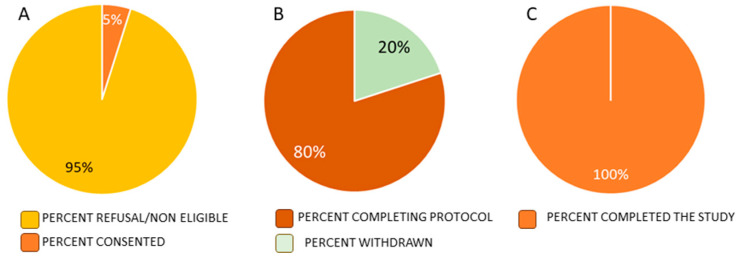
Consent rate. Recruitment (**A**). We approached 205 potential participants, but only ten individuals were eligible for study enrolment and randomization, for a consent rate of <5% (10/205); Retention (**B**)—withdrawn for safety reasons (transaminitis and aggravation of pre-existing anemia due to frequent blood draw); and Percent that completed the study (**C**).

**Figure 4:**
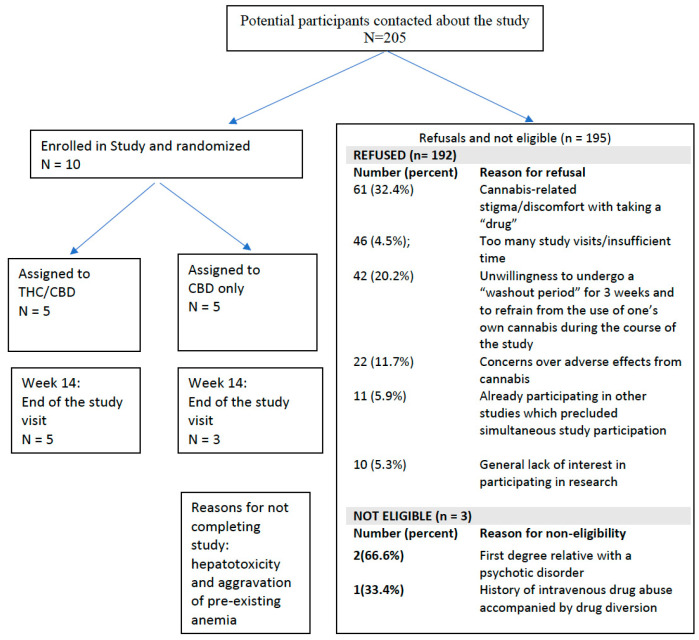
Recruitment Summary.

We approached 205 potential participants. Ten were consented and randomized for a consent rate of 5%. There were 195 individuals that refused or were not eligible. In four cases out of seven, individuals did not agree to undergo a washout period of their current cannabis product and did not believe they could undergo a washout due to favorable effects of cannabis on their chronic pain or mood, presenting a barrier to recruitment. One of these individuals indicated that a barrier to study participation was that it can take nearly a year for approval for reimbursement from a government reimbursement program to access CBD for free and participation in this study would halt access to CBD through this reimbursement program. In three cases out of seven, individuals had contra-indications to study enrolment which were not detected via chart review screening. Therefore, only three individuals were eligible for study enrolment and seven were enrolled with minor protocol deviations as shown in Figure 3. Reasons for low recruitment numbers are presented in Figure 4.

As this pilot study explored a phenomenon with few in vivo data, a convenience sample of 26 participants was chosen as the target sample size without formal power calculations [7]. This number of participants was deemed sufficient to assess feasibility (willingness of individuals to participate, attend study visits and adhere to study procedures), assess numbers of dropouts and evaluate safety and tolerability [7]. Although the small number of participants may result in wide CIs for AEs, this number of participants should give us an idea of variability for continuous outcomes to assist in power calculations for future studies [7].

Characteristics of the 10 participants enrolled in the study were previously published; eight (80%) were male, six (60%) were white and all were virally suppressed on ART [29]. The majority of participants were cannabis-experienced and seven (70%) had used cannabis in the past 6 months [29]. Of the participants endorsing cannabis use in the past 6 months, five (72%) indicated monthly use, two (29%) weekly use and none indicated daily use. Many participants already used cannabis regularly for chronic pain (neuropathic pain, pain due to arthritis related to hemarthrosis from hemophilia and menstrual pain) and to reduce stress. Many participants who joined the study expressed an interest in doing so in order to see whether the capsules could relieve symptoms of chronic pain.

### 3.2. Compliance

Enrolment compliance was 30% as seven participants were enrolled with minor protocol deviations. Of the 10 individuals randomized, all participants followed study procedures as described. Compliance with data collection and adherence to study procedures were excellent with 100% of visits attended within the study window and 100% of daily diary and questionnaires completed. Adherence to daily capsule consumption and the titration protocol was excellent. There were no concerns voiced by participants or study staff regarding the patient diary.

### 3.3. Retention

Although 10 individuals were randomized, two individuals were withdrawn for safety reasons. As previously reported in detail, one individual experienced severe adverse effects with hepatotoxicity deemed to be multifactorial in nature. The other individual was by withdrawn after 6 weeks of CBD due to aggravation of pre-existing anemia due to frequent phlebotomy. Eight participants were retained until the end of the study and were highly engaged throughout the study period, providing a retention rate of 8/8 (100%).

### 3.4. Study Staff and Team Feedback

Co-investigators, other MDs (both clinic physicians and study sub-investigators) and some study staff at the clinic were surprised that participants were not selected based on a specific indication for cannabinoid use, such as chronic pain or anxiety. From a study staff perspective, clearer exclusion criteria were requested for some items, such as liver disease, where specific transaminase ranges were requested. Furthermore, we revised our exclusion criteria to include individuals with end-stage renal disease or CrCl < 30 mL/min or requiring hemodialysis, rather than <90 mL/min, since many PWH are aging and may have reduced renal function. Cannabinoids are predominantly metabolized by the liver and only a small minority of inactive metabolites are excreted in the urine [35,36,37]. A summary of changes and amendments made throughout the course of the study, along with the rationale, are presented in Table 2.

Changes made in response to recruitment challenges: Mid-way through study recruitment, we re-assessed whether we could reduce the overall number of study visits. However, given the concern related to using CBMs, and especially for severe hepatotoxicity, as was observed in one person and mild transaminitis in another, the study team felt it prudent to continue to have participants perform safety blood draws every 2 weeks. We also reached out to four community HIV clinics regarding whether they had patients who were potentially interested in participating, but this did not result in recruitment of additional participants.

### 3.5. Participant Feedback

At the final study visit, participants were asked for general feedback about the study. Although study visits were frequent, most understood the need for close safety monitoring in a pilot study involving cannabinoids. Participants who enrolled all agreed to take the cannabinoid capsules and did not express concern with the intervention. The titration protocol worked well, and participants and study staff understood that participants should titrate dosing to their level of comfort. Therefore, when a participant experienced excessive daytime somnolence, the capsule was taken in the evening before bed. This flexibility was beneficial as it enabled participants to remain in the study as long as they were taking at least one dose of cannabinoids daily. With regards to specimen collection, participants did not express any concern with providing a stool sample, and all the males in the study agreed to the optional sub-study involving provision of a semen specimen pre and post study intervention. Participants also felt that the amount of study compensation (CAD 25 per visit) was appropriate.

Some participants were surprised that the combined THC/CBD capsules did not induce a sense of euphoria common with smoked cannabis, and some even experienced worsened mood [29]. Some participants with chronic pain who were used to smoking cannabis missed the rapid onset of action with smoking, compared to the slower onset of action with the capsules. As we previously reported, the cannabinoids did not result in any significant changes in quality of life or mood measures [29].

Some participants enrolled had different experiences throughout the study which they shared with us in an interview setting when asked about how their participation in this study impacted them day-to-day. A couple of participants found that the trial positively impacted their quality of life; however, dosage was important.

“It helped me to fall asleep much better and be asleep longer. Without cannabis, I usually only sleep for three hours and then I can’t go back to sleep anymore. Using cannabis also helped with my restless leg syndrome, but it didn’t help me with my appetite. My first dosage for the trial was perfect, but after that the dosage doubled, I was kind of knocked out and I thought I better not drive. I would fall asleep in the middle of the day at my desk, so I think the first dosage was the best for me.”

Participant 1

“[Participating in this trial] did wonders for me! I felt like my anxiety wasn’t as severe because I do suffer from anxiety. And I did feel very good. It helped me rest and improved my appetite too. I also have trouble with my bowels from my medication and it helped make me feel far more comfortable. I wouldn’t want to go any higher than what I was provided because for me it was a perfect dose. After the trial stopped, I didn’t experience any withdrawals symptoms, but I just wished that I could continue because it was doing so much good for me.”

Participant 2

There were also participants who felt that the trial did not benefit them, one of whom found that two doses negatively impacted their ability to work safely.

“I did feel a little bit tired, sleepy. With the first dose, it wasn’t that terrible, but with two doses, my drowsiness got worst. I felt really sleepy and, in the morning, sometimes I would feel a little bit nauseous and I couldn’t focus at work. I told the nurse about how I was feeling and they said that I could switch back to the first dose. I didn’t find that the cannabis did anything for me. Even though I live with HIV, my health situation is really good. I don’t have pain or depression so participating in this trial was a little bit negative because I didn’t like feeling sleepy.”

Participant 3

“I’ve been trying to something to help me with the pain in my legs, because after I had chemotherapy, my feet always feel something. Either tingling or pain or like someone is touching them, but nothing is actually happening to them. I joined the trial because I always want to try something so that I don’t feel it anymore. The cannabis that I tried, it made me paranoid, like people are watching me, but it didn’t do anything for my feet.”

Participant 4

One participant also emphasized that, even though he did not personally benefit from participating in the study, he recognized the potential benefit of this work for others, especially for those who use cannabis as an adjunct to their medication.

“It did not help me at all but I enjoy volunteering for studies, because even though it might not help me, it might help others. I also wanted to be in this study because I try to use everything natural, and cannabis comes from a natural plant. Even though it did not help me, and I am not using it, I am pro-cannabis. I think if something is good for someone, if it’s working for many people, why stop people from having it?”

## 4. Discussion

Pilot studies are effective in reducing waste in medical research by assisting researchers in identifying how they can adjust study procedures and reduce waste. Pilot studies are especially useful in fields where participants are difficult to recruit and retain, such as in HIV research [38]. Feasibility assessments help determine region- or institution-specific practices which impact study completion [38]. In the current pilot clinical trial, we evaluated the safety, tolerability and feasibility of administering oral cannabinoid capsules to PWH, with feasibility outcomes as the focus of the current manuscript. In addition to the timeliness of initiating this clinical trial, feasibility outcomes included consent rate, retention and compliance, along with verbal feedback solicited from participants and the study team. We report on factors impacting the timeliness of study initiation, including changing cannabis legislation within Canada during the study period, and the impact of the COVID-19 pandemic on non-COVID-19-related research. As we were unable to enroll sufficient participants, this meant that we could not confidently make conclusions about the effects of the cannabinoids on markers of inflammation, gut microbial translocation and reservoir size. Although trends may have been observed, we must be cautious in interpreting the findings due to the small sample size. Our primary objectives (safety and tolerability) could only be examined in a small number of individuals. Furthermore, we had originally wanted to examine the outcomes stratified by treatment group (i.e., CBD-only or THC/CBD combined capsule). Because of the small sample size, we decided to group all of our participants together.

The two largest barriers in this study were timeliness to study initiation and completion and challenges with recruitment. Globally, more than 80% of trials are unable to enroll on time, requiring study extension and potentially addition of new sites [39]. A major barrier to beginning the current study was the requirement to obtain a Cannabis Research License, which resulted in an 11-month delay from the time of application to the time we received the license from Health Canada. As researchers must wait in the same queue as licensed producers when seeking approvals from Health Canada, this may result in lengthy waiting times and delaying study initiation [40]. We have previously reviewed changes in the Canadian legislation landscape and their impacts on cannabis and cannabinoid research [40]. The COVID-19 pandemic also had an impact on the timeliness to study initiation, requiring us to amend the protocol to enable remote follow-up when feasible. It has been reported that more than 40% of clinical trials amend their protocol before the first participant visit, resulting in a delay of 4 months from protocol implementation to participant enrolment [41].

Recruitment was the most challenging aspect of conducting this study and we were unable to include more than 10 participants in this pilot study, and therefore we did not reach our first target sample size. This is consistent with what is reported in the literature for clinical trials, in which 35% of delays in conducting clinical trials are attributed to delays in recruitment [42]. In a review of all studies completed between 2014 and 2018 at a center in Mumbai, India, Bose et al. evaluated four pre-identified criteria for screen failures and dropouts: risk, nature of funding, study design and nature of participants [43]. They reported that high-risk and interventional studies were predictors for both screen failures and dropouts, whereas pharmaceutical industry-funded studies and healthy participants were predictors for only screen failures [43]. In a comprehensive review of RCTs funded by the United Kingdom National Institute for Health Research (NIHR) and published in the online NIHR Journals Library between January 1997–December 2020, Jacques et al. identified 388 RCCTs and 379 reports in the NIHR Journals Library [44]. They found that the final recruitment target sample size was achieved in 63% of the RCTs and that the original recruitment target was revised in 30% of trials (downwards in 67%) [44].

Studies involving CBMs require additional feasibility considerations given that cannabis/CBMs have additional regulatory requirements and may be associated with stigma. In Canada, the federal government considers cannabis/CBMs controlled substances, meaning that they are categorized as having a higher-than-average potential for abuse or addiction. Such drugs are divided into categories based on their potential for abuse or addiction [45]. Status as controlled substances results in additional regulatory considerations, contributing to more paperwork, and the need for particular storage and handling by the pharmacy and team [46].

Relatively few pilot feasibility studies involving CBMs have been conducted. To our knowledge, none of the feasibility pilot studies involving CBMs have been in Canada nor in PWH. The populations in the CBM feasibility studies were very diverse and included individuals with long COVID (United Kingdom) [47], cancer patients with cancer-related symptoms (Australia) [48], dialysis patients with chronic pain (Israel) [49], individuals with crack use disorder (Brazil) [50] and children with autism spectrum disorder (ASD) (Israel) [51]. In a single-arm, open-label feasibility trial in the United Kingdom of the safety and tolerability of a full-spectrum CBD-dominant CBM product for treating symptoms of long COVID, Thurgur et al. treated 12 participants with up to 3 mL of 5% CBD Oil (50 mg CBD/mL, <2 mg THC/mL) per day orally for a total of 21 weeks, followed by ~3 weeks without the study drug [47]. Participants adhered to the treatment protocol, there were no serious adverse events and response rates for the research assessments were high (>90% completion of patient-reported outcome measures and daily self-report). However, they also experienced challenges related to recruitment strategy and their initial proposal to recruit participants was amended [47]. Meanwhile, Good et al. conducted a prospective two-arm open-label pilot trial of escalating doses of CBD and THC oil in participants with advanced cancer and cancer-related symptoms in an Australian palliative and supportive care service of a cancer center to assess the feasibility of using global symptom burden measures to assess response to medicinal CBMs, to determine median tolerated doses of CBD and THC and to document adverse events [48]. One of the main outcome measures was the number of participants screened and randomized over the time frame and the number of participants completing days 14 and 28 and providing total symptom distress scores (TSDSs) [48]. Of the 21 participants enrolled (CBD, *n* = 16; THC, *n* = 5), 18 (86%) completed the primary outcome measure on day 14 and eight completed day 28 [48]. In another randomized trial of medical cannabis in patients with stage IV cancers in Israel to assess feasibility, dose requirements, impact on pain and opioid use, safety and overall patient satisfaction, Zylla et al. randomized 30 participants 1:1 to early cannabis (EC, *n* = 15) versus delayed start cannabis (DC, *n* = 15) [52]. The EC group obtained 3 months (3 M) of CBM through a state program at no charge, while the DC group received standard oncology care without CBMs for the first 3 months [52]. Participants met with licensed pharmacists at one of two CBM dispensaries to determine a suggested CBM dosing, formulation and route. Patients completed surveys on pain levels, opioid/MC use, side effects and overall satisfaction with the study [52]. In contrast to our study and that by Thurgur et al., Zylla et al. were more successful with recruitment as 36% of patients who met the eligibility criteria ultimately enrolled [52]. A higher proportion of EC patients achieved a reduction in opioid use and improved pain control [52].

The feasibility of CBMs has also been examined in studies involving different patient populations. In another feasibility study in Israel, Kliuk-Ben Bassat et al. examined feasibility and safety of sublingual oil-based medical cannabis for chronic pain management in hemodialysis (HD) patients [49]. In this prospective randomized, double-blind, cross-over design, participants were assigned to one of three arms: BOL-DP-o-04-WPE whole-plant extract (WPE), BOL-DP-o-04 cannabinoid extraction (API) or placebo. WPE and API contained THC and CBD in a 1:6 ratio (1:6, THC:CBD) [49]. Participants were treated for 8 weeks, with a subsequent 2-week washout, followed by a cross-over to a different arm. The primary endpoint was safety. Out of 18 patients recruited, 15 were randomized. Three did not complete the drug titration period due to AEs and one patient died during titration due to sepsis (WPE). Of those who completed at least one treatment period, seven patients were in the WPE arm, five in the API and nine received placebo [49]. Most AEs were mild to moderate and resolved spontaneously. In contrast to our study, liver enzymes were stable during cannabis treatment for all participants. Thus, they concluded that short-term medical cannabis use in patients treated with HD was generally well tolerated, and the investigators concluded that the safety data support further studies to assess the overall risk–benefit of a treatment paradigm utilizing medical cannabis to control pain in this patient population [49]. Feasibility has also been examined in persons with crack use disorder and in pediatrics for children with autism spectrum disorder (ASD). In a retrospective study, Aran et al. assessed the tolerability and efficacy of cannabidiol-rich cannabis in 60 children with ASD and severe behavioral problems (age = 11.8 ± 3.5, range 5.0–17.5; 77% low functioning; 83% boys) [51]. Efficacy was assessed using the Caregiver Global Impression of Change scale. Adverse events included sleep disturbances (14%), irritability (9%) and loss of appetite (9%). One girl who used higher THC had a transient serious psychotic event which required treatment with an antipsychotic. Following the cannabis treatment, behavioral outbreaks were much improved or very much improved in 61% of patients. The investigators concluded that their preliminary study supports the feasibility of CBD-based cannabis trials in children with ASD in Israel [51]. However, these findings may not be applicable to other countries and researchers must take into account the regulatory environment surrounding CBMs, literacy of the target population and issues surrounding stigma with the use of CBMs for medicinal purposes. Both healthcare providers and the public in Israel may be more open toward CBMs than physicians and the public in other countries [53,54].

The COVID-19 pandemic was a factor impacting timeliness to study initiation and recruitment, as many individuals did not want to come to the clinic, based in a tertiary care center, more than necessary in order to limit exposure. It is possible that PWH perceived themselves to be at greater risk of COVID-19 acquisition due to being considered “immunocompromised”, at older age and with a large burden of comorbidities. During the pandemic, PWH in Canada reported frequent practices of preventative behaviors including masking, physical distancing and limiting contact with at-risk individuals [55] and thus it is plausible recruitment was impacted due to the frequent study visits. As our clinic is very active in research in clinical trials and other research studies, it is possible that eligible participants may have been interested in our study but had already committed to being enrolled in another study, precluding participation. In a review of critical issues and challenges in the recruitment and retention of participants in clinical studies, Desai also comments on the competition for potential patients with multiple studies simultaneously recruiting participants at the same center [39]. In discussions with some study staff at our site, the view was expressed that studies focused on antiretroviral medications should take higher priority given the need of all PWH to take these medications and that studies for cannabis, while valid, would not have the same priority level. When initiating this study, there was discomfort related to cannabis’ classification as a controlled substanceat both the staff and study staff level, as well as amongst some PWH approached for potential participation. In a survey of Canadian healthcare providers, many were unfamiliar with topics related to CBMs and uncomfortable due to the lack of pre-existing clinical trials for efficacy and safety [39]. Ongoing work is also required to reduce the stigma associated with CBMs.

Differences across feasibility studies should also take into account different definitions used. Although numerous articles describe guidelines for the conduct of clinical studies, few have included specific feasibility indicators or strategies for evaluating multiple aspects of feasibility [56]. In their review, Teresi et al. present examples of indicators (recruitment, retention, intervention fidelity, acceptability, adherence and engagement) for feasibility assessment of data collection methods and intervention implementation and underscore the importance of examining feasibility when adapting an intervention tested in mainstream populations to more diverse groups [56]. It must be underscored that current feasibility studies use different definitions to define indicators, which makes comparison across studies challenging. For example, the recruitment rate may be defined as the proportion of all eligible participants who are enrolled/randomized or the proportion of participants approached who agree to undergo enrollment/randomization. Similarly, retention rate may be defined as enrolled participants with valid primary outcome data or the proportion of enrolled participants who are present throughout the length of the study until the final study visit [44,47,48,56].

The participants we did enroll in our study are not necessarily representative of many of our clinic patients, nor of the HIV epidemic in Canada. In our study, the majority of participants did not hold regular employment, 80% were male and 60% were white North Americans. In contrast, 40% of our clinic clients are Caucasian [57] whereas in Canada, there are increasing cases of HIV amongst women and non-Caucasian migrants [58]. In the aforementioned pilot feasibility study on CBMs for individuals with long COVID, Thurgur et al. also had a participant pool which was somewhat homogeneous and consisted mostly of white British women [47]. Collecting reasons for non-enrollment may help to determine what factors could be improved in future studies and to determine how participants enrolling in the study differ from the general clinic or patient population.

This study was resource-intensive. Due to significant delays in study initiation and roll-out, the costs associated with the study were much higher than calculated (mainly due to costs associated with study staff). There were delays associated with hiring and training of new staff as the study start was delayed and increased costs associated with amending protocols and ICF to account for activities which could be carried out remotely during the COVID-19 pandemic. Staff found the frequency of study visits onerous but recognized their importance for monitoring purposes, especially in the context of transaminitis. Participants that experienced unexpected adverse events attended additional study visits for follow-up There were also costs associated with pharmacist consultations and additional record-keeping required for cannabis as a controlled substance. Physical space requirements in the pharmacy must also be taken into account since bottles of capsules must be stored in locked cabinets. For this study, we obtained regulatory support as an in-kind contribution from the CTN. However, costs associated with submissions to Health Canada, including all protocol amendments, must be factored into the budget for studies involving cannabinoids. Moreover, participants who were using cannabinoids regularly prior to study enrolment found it difficult to stop using cannabis, especially when they were achieving positive effects for symptom control. Some participants who found the study capsules helpful expressed disappointment at not being able to access the study capsules following study termination.

The summary of recommendation for future studies, based on our experience, can be found in Table 3.

## 5. Conclusions

Recruitment was the most challenging aspect of conducting this study, with a low consent rate. Ongoing work is required to reduce stigma related to CBMs. Future studies should find a balance between requirements for safety monitoring and frequency of study visits. Interestingly, the need to refrain from using one’s own cannabis and to undergo a washout period was a major factor precluding participation for many individuals approached. Plans to enable participants to experience beneficial effects from study interventions should be in place to enable them to obtain the treatments following study completion. Information obtained from pilot studies is helpful to inform the design and implementation of large-scale studies, especially for non-conventional therapies with complex regulatory statuses.

## Figures and Tables

**Table 1:** Barriers and facilitators to conducting the CTNPT 028.

Study Barriers	Study Facilitators	Study Limitations	Study Strengths
1. Changing Legislation in Canada → Cannabis Research License; 2. Restrictive inclusion/exclusion criteria; 3. COVID-19 pandemic; 4. Poor consent rate/recruitment challenges (frequent study visits, stigma, competing priorities); 5. Short capsule expiry dates; 6. High costs associated with cannabis capsule manufacturing and unforeseen supplementary visits for SAE.	1. High level of community enthusiasm; 2. Excellent participant engagement, retention and compliance.	1. Possible selection bias? (Nearly all participants were cannabis-experienced or regular cannabis users for pain or anxiety.) Tendency to approach participants known for cannabis use.	1. Support of the CTN National Centre with regulatory experience and Health Canada submissions; 2. Site experience with running clinical trials and access to specialists for support (hepatologists, etc.); 3. Study materials: the participant dosing diary was effective for keeping track of capsule intake and titrations, adverse effects, and easy to use.

**Table 2:** Main protocol changes after study initiation based on study staff and participant feedback.

Protocol Changes	Rationale
Protocol V4.1, 20 May 2021 toV5 28 October 2021	
Change to exclusion criteria: Removed “On antipsychotic medication”.	Removed “On antipsychotic medication” because some people use these medications for sleeping, i.e., Seroquel may be listed as an antipsychotic but many people without psychosis use it for sleep.
Change to exclusion criteria: Revised to exclude participants with end-stage renal disease (defined as creatinine clearance < 30 mL/min) or requiring hemodialysis).	Many people are aging and may have reduced liver function. Hemodialysis, rather than renal dysfunction defined as creatinine clearance < 90 mL/min.
Change to exclusion criteria: Defined active liver disease as current diagnosis of cirrhosis, hepatic encephalopathy, alcoholic liver disease, primary biliary cirrhosis or primary sclerosing cholangitis, autoimmune hepatitis, Wilson’s disease, hemochromatosis or iron overload or alpha-1 antitrypsin deficiency and clarified that non-alcoholic fatty liver disease is not an exclusion criterion.	There is no evidence to suggest that persons with non-alcoholic fatty liver disease (NAFLD) will metabolize cannabinoids less efficiently than persons without NAFLD.
Protocol V5, 28 October 2021 to V 11 April 2021	
Change in inclusion criteria: The washout period for stopping cannabis was reduced to 1 week from 3 weeks.	Three weeks is the complete washout period for cannabis. However, participants report discomfort in being off cannabis for such a long period, which has significantly impacted recruitment. When cannabis consumption is stopped for 1 week, the levels left in the body have declined substantially so that they are not likely to impact markers of inflammation or reservoir size.
Change in inclusion criteria: A negative urine cannabis screen is not required to participate in the study.	A person may have a positive cannabis urine screen yet still be enrolled in the study since this test will detect residual cannabis in the body. As indicated above, cannabis in such low levels is unlikely to affect inflammation or reservoir size. The cannabis urine test will be continued to be performed for documentation, since it may be helpful when interpreting study results.
Change in exclusion criteria: Modified to exclude participants endorsing binge alcohol drinking on the AUDIT screen.	Alcohol can increase liver enzymes and, when combined with CBD, may increase hepatotoxicity. Participants may have a negative urine alcohol screen if they do not drink close to the study visit; however, they may still have problematic drinking. Participants with a positive AUDIT screen will be excluded, even if the urine alcohol screen is negative.
The maximum daily dose of CBD in Group 2 was reduced to 400 mg daily (previously 800 mg daily).	No available data on the maximum daily doses of CBD which are safe for the HIV population. In studies on Epidiolex in patients with epilepsy, CBD daily doses were 800 mg/day and transaminitis was observed in 13% of patients. However, as people with HIV have high levels of baseline liver disease, a lower maximum dose of CBD may be prudent.
A guideline for managing increased liver enzymes is included within the protocol.	This was added to enhance study safety. Changes included specifying actions required based on transaminase levels.

**Table 3:** List of recommendations for future studies involving cannabis and cannabis-related products.

Recommendation	Reasoning
Start with a pilot study.	Feasibility assessments help determine the region or institution-specific practices which will impact study completion, enrolment and retention of participants, safety issues.
Advocate for expansion of the regulatory requirements or regulate the study under Institutional Research License.	Cannabis products manufactured under Good Manufacturing Practices (GMPs) are not readily available. Commercial cannabis, purchased and used by patients and consumers in the “real-world” setting, is manufactured under Good Production Practices (GPPs), the current standard required under Part 5 of the Cannabis Regulations. Since the cannabis industry is not required to partake in the standard drug approval process when seeking approval for medical use, there is little incentive to support research to have their products approved for the medical market.
Reduce the number of visits to the minimum necessary while still being mindful of safety and the possibility of abuse of large quantities of cannabis provided for the study.	The frequency of study visits was the second most common reason for declining participation for many individuals. This study overlapped with many COVID-19 pandemic measures; in-person visits were even harder to complete.
To recruit a representative population for your study, increase advertising and involve the community in preparing and distributing advertising material. Women are especially difficult to recruit and keep in the study as they are often sole care providers for their family, and are more affected by stigma.	In our study, eight (80%) of our participants were male and six (60%) were white North American, which does not reflect the demographics of our clinic population.
Involve and consult pharmacists in protocol preparation and participant follow-up.	Because of drug interactions, several pharmacy consultations and adjusted recommendations for medication modifications had to be implemented to avoid drug interactions with cannabinoids.
Work on destigmatizing the use of cannabis for medical purposes and clearly state the possible benefits to participant problems.	When initiating this study, there was discomfort amongst both the clinic and study staff, as well as amongst several potential participants, in conducting and participating in cannabis research. Targeting recruitment for participants with a specific medical problem (i.e., insomnia, chronic pain) or conditions might facilitate recruitment.
Develop a standard end-of-study questionnaire. Prepare end-of-study or exit interviews with participants.	We performed informal open discussions about the overall impressions of the study with participants. A more structured and uniform questionnaire would provide systematic data that can be used for improving future studies. Partner with experts in patient-oriented outcomes research to develop questionnaires and interview questions tailored for cannabinoid-based medicine research.

## Data Availability

Data are contained within the article.
